# Time series analysis of suicide from a monthly perspective in the south of Brazil: an ecological study

**DOI:** 10.47626/2237-6089-2021-0202

**Published:** 2021-11-09

**Authors:** Augusto Mädke Brenner, Felipe Cesar de Almeida Claudino, Gianfranco Rizzotto de Souza, Neusa Sica da Rocha

**Affiliations:** 1 Universidade Federal de Ciências da Saúde de Porto Alegre Faculdade de Medicina Porto Alegre RS Brazil Faculdade de Medicina, Universidade Federal de Ciências da Saúde de Porto Alegre (UFCSPA), Porto Alegre, RS, Brazil.; 2 Hospital de Clínicas de Porto Alegre Centro de Pesquisas Clínicas Porto Alegre RS Brazil Centro de Pesquisas Clínicas, Hospital de Clínicas de Porto Alegre (HCPA), Porto Alegre, RS, Brazil.; 3 Universidade Federal do Rio Grande do Sul Innovations and Interventions for Quality of Life Research Group (I-QoL) Porto Alegre RS Brazil Innovations and Interventions for Quality of Life Research Group (I-QoL), Universidade Federal do Rio Grande do Sul (UFRGS), Porto Alegre, RS, Brazil.; 4 UFRGS Programa de Pós-Graduação em Psiquiatria e Ciências do Comportamento Porto Alegre RS Brazil Programa de Pós-Graduação em Psiquiatria e Ciências do Comportamento, UFRGS, Porto Alegre, RS, Brazil.; 5 UFRGS Departamento de Psiquiatria Porto Alegre RS Brazil Departamento de Psiquiatria, UFRGS, Porto Alegre, RS, Brazil.

**Keywords:** Suicide, completed suicide, epidemiology, mental health

## Abstract

**Introduction:**

Suicide is the cause of death of almost 800 thousand people worldwide every year. In Brazil, Rio Grande do Sul is one of the states with the highest suicide rates. This study aimed to assess whether there is a significant monthly time pattern of suicide in Rio Grande do Sul, by gender and age ranges, and whether suicide characteristics in the state are coherent with findings from previous studies.

**Methods:**

All data were collected from official secondary sources maintained by the national Brazilian and Rio Grande do Sul governments, covering a period from 2015 to 2019. Data included suicide deaths and population, divided by gender and age range. Sum totals, frequencies, odds ratios, and time series analyses were performed.

**Results:**

From 2015 to 2019, 6,287 people committed suicide in Rio Grande do Sul. Most of them were men and the most prevalent age band was from 50 to 59 years old. Men had higher suicide rates then women in all age ranges (p < 0.001) and in all months of the year, with an approximately 4-fold higher risk of committing suicide when compared to women. Men had a trending peak of suicide in January and December (p < 0.001), whereas women’s suicide rates peaked in March and December (p = 0.001).

**Conclusion:**

There are monthly time trends and seasonal patterns of suicide rates in Rio Grande do Sul, varying by gender and age range. Gender differences occurred mainly in the first three months of the year, and the age pattern was more evident among individuals aged 60 years or older.

## Introduction

Suicide is a global public health issue, being the cause of death of almost 800 thousand people every year.^1^ The World Health Organization (WHO) estimates that for every suicide there are more than 20 other suicide attempts.^2^ Several factors seem to be associated with this phenomenon, including neurobiological factors,^3^ such as serotonergic abnormalities and inflammatory mechanisms, and sociodemographic factors,^4^ such as age, gender, and education, with 79% of global suicides in 2016 occurring in low and middle-income countries.^1^ The most common methods of suicide include ingestion of pesticides, hanging, and firearms, but the choice often varies according to population group.^5^ There are also differences between genders when attempted and completed suicides are compared: higher rates of suicide attempts have been described in women,^4^ whereas higher rates of completed suicide have been observed in men.^6^

In Brazil, from 2011 to 2017, there were 80,352 suicide deaths registered in the population over 10 years old, 27.3% of which occurred in the age range from 15 to 29 years of age and the vast majority in men.^7^ The increase in suicide rates has been evenly distributed across the country, but intentional self-harm notifications were centered in the states of São Paulo (SP), Minas Gerais (MG), Paraná (PR), and Rio Grande do Sul (RS).^7 , 8^ In an epidemiological study of suicide in Brazil from 1980 to 2006, Lovisi et al.^8^ showed that the south region, which comprises the states of RS, Santa Catarina (SC), and PR, had the highest suicide rates in the country. In addition, they identified Porto Alegre, state capital of RS, as the Brazilian capital with the second highest suicide rates. In another Brazilian study, Rodrigues et al.^9^ found that suicide rates for both genders were highest in RS.

There are studies describing an apparent annual distribution of suicide, with rates peaking in late spring and early summer.^10^ Different approaches to analysis of monthly distribution have been proposed, considering seasons or specific dates of the year, such as national holidays.^11^ However, there is still a lack of data on the characteristics of suicide trends and seasonality in the current literature, such as the differences in seasonal trends between genders and age ranges. In addition, few studies have described analyses of suicide from a monthly perspective since most investigations have focused on year-over-year trends and forecasting. Furthermore, few studies have analyzed monthly suicide trends and seasonal features in South America, and even fewer in the south of Brazil.

Considering that suicide rates are often underreported, even in countries with good vital statistics data, better comprehension of the factors associated with suicide could help enhance protective factors and related interventions, since suicides are considered preventable deaths.^5^ In consonance with the importance of suicidology and the understudied high rates of suicide in RS, regional studies may provide a better and more focused view of suicide characteristics and distributions throughout the year. Our aim was to investigate whether there are significant monthly time trends and seasonal patterns of suicide rates in RS by gender and age range and whether the state’s suicide rates and characteristics are coherent with previous findings reported in the literature. Our hypothesis is that suicide mortality in RS is more prevalent among men and older individuals, with deaths peaking in the spring and summer months of the year, and that seasonality has different impacts across genders and age ranges.

## Materials and methods

### Study design and data source

In this ecological study, we describe data on suicide rates and population obtained from official secondary sources maintained by the national Brazilian and RS governments and stored in information systems. Our data set comprises public information on suicide and demographic data from the state of RS covering the period from 2015 to 2019.

All data described in this article were collected in aggregate form. Data regarding number of suicides in total, by age range (less than 10, 10 to 19, 20 to 29, 30 to 39, 40 to 49, 50 to 59, 60 to 69, 70 to 79, and over 80 years), and by gender were obtained via the RS State Health Surveillance Center (CEVS). The CEVS provides the most recent public data from databases maintained by the Brazilian Ministry of Health ( http://bipublico.saude.rs.gov.br/ ). Since we noted that suicide numbers were concentrated at higher age ranges, age stratification was performed to better evaluate correlations with gender and monthly time trends. Age ranges were stratified as less than 20 years old, 20 to 39 years old, 40 to 59 years old, and 60 years or older. Demographic data for the total estimated 2019 population and divided by gender and age range, as addressed before, were collected via the Brazilian Institute of Geography and Statistics (IBGE). All data were collected from May 22, 2020 to May 30, 2020. According to the 10th Revision of the International Classification of Diseases (ICD-10), suicide is defined as death resulting from intentional self-harm, and codes X60 to X84 and Y87 are used to identify this outcome.^12^

### Statistical analyses

Demographic data were described using sums and frequencies. We present suicide rates as totals, by age, and by gender, per 100,000 population. Comparisons between genders (men/women) in total and for each age strata were performed using chi-square tests. Normality was assessed with the Shapiro-Wilk test. Only suicide rates for all females and for people less than 20 years old of both genders were found to be non-parametric. However, since the results did not differ when these samples were analyzed with non-parametric tests, we conducted all analyses with parametric tests. The influence of age range on suicide rates was assessed using linear regression models. Odds ratios were calculated for gender and between the age ranges with highest and lowest frequencies of suicide, in total, and broken down by gender. A 95% confidence interval (95%CI) was adopted for odds ratios.

Our primary statistical goal was to use descriptive and explanatory models to analyze our data, not to predict or forecast future events. Therefore, we used regression models to observe trends and seasonality in our time series analysis. Our objective was to assess these data on suicide deaths and determine whether gender and age ranges had significant impacts on values. At first, all data were analyzed using a linear regression model. However, since suicide deaths are not linear throughout the year, a linear regression model may systematically underestimate and/or overestimate the values.^13^ Therefore, in order to fit the deterministic trend seen in our data, we utilized higher-order polynomial models, such as quadratic and cubic functions. Model fitting was established first by p-value and second by R^2^ among significant models. Since data comprise a seasonal time series component, a repeating pattern occurs over time. As our objective was to assess seasonality, adjustment for seasons was not required. Odds ratios between genders in January and December were calculated as a post hoc analysis, considering our findings and objectives. All data were considered statistically significant at p < 0.05. Statistical analyses were performed using the SPSS version 26.0 software package (IBM Corporation, Armonk, NY, USA).

### Ethical considerations

Since this study only involves secondary data from an official Brazilian Ministry of Health database source, it was exempt from evaluation by a research ethics committee, in accordance with Brazilian National Health Council Resolution number 466 from December 2012.

## Results

### Gender and age range: population features of suicide rates

The Brazilian Mortality Information System registered 6,287 cases of suicide in RS from 2015 to 2019, mostly men (79.67%) and with greatest prevalence from 50 to 59 years of age (19.85%). We found a statistically significant difference between genders in suicide mortality (X^2^ (1, N = 6,287) = 69,941, p < 0.001). Men had higher percentage rates of suicide when compared to women, and this finding extends to all age ranges, as seen in Table 1 . Regarding the gender ratio for overall suicide mortality, we found that men committed suicide 3.92 times more frequently than women. The younger than 20 years old and 40 to 59 years age ranges had lower gender ratios than the overall ratio. In contrast, the 20 to 39 years old and 60 years or older age ranges had higher gender ratios than the overall ratio ( Table 1 ). We also found that older age ranges were associated with higher frequencies of suicide deaths ( Table 1 ).

**Table 1:** Demographic and suicide data (2015-2019)

Variable			Deaths by suicide	Gender ratio (men/women)	Population	p-value *
Age (n = 6,284), n (%)						
	Under 20 years		324 (0.01)	2.90	3,075,396	**< 0.001**
		Men	241 (0.015)		1,573,354	
		Women	83 (0.005)		1,502,042	
	20 to 39 years		1,820 (0.05)	4.30	3,477,118	**< 0.001**
		Men	1,477 (0.084)		1,754,490	
		Women	343 (0.020)		1,722,628	
	40 to 59 years		2,340 (0.08)	3.50	2,934,846	**< 0.001**
		Men	1,820 (0.128)		1,425,633	
		Women	520 (0.034)		1,509,213	
	60 years or older		1,800 (0.10)	4.42	1,760,563	**< 0.001**
		Men	1,468 (0.192)		765,874	
		Women	332 (0.033)		994,689	
Gender (n = 6,287), n (%)						
	Men		5,009 (0.09)	3.92	5,519,351	**< 0.001**
	Women		1,278 (0.02)		5,728,572	
Rio Grande do Sul			6,287		11,247,923	

* Differences between genders were evaluated with chi-square tests.

Comparing suicide rates by gender, we found that men had an odds ratio of 4.07 (95%CI = 3.83-4.33) when compared to women. Regarding age ranges, we found that people aged 60 years or older (higher suicide rate) had an odds ratio of 9.71 (95%CI = 8.63-10.93) when compared to people less than 20 years old (lower suicide rates). Also, men aged 60 years or older (higher suicide rate) had an odds ratio of 12.54 (95%CI = 10.94-14.36) in comparison with men less than 20 years old (lower suicide rates). For women, those 40 to 59 years old (higher suicide rate) had an odds ratio of 23.13 (95%CI = 18.56-28.82) when compared to those less than 20 years old (lower suicide rates).

### Gender and age range: monthly trend and seasonality of suicide

When we analyzed the monthly distribution of suicide rates by gender, we found different seasonal patterns of suicide deaths between men and women. As shown in Table 2 , men had a consistently higher suicide rate than women all year long.

**Table 2:** Means and standard deviations for monthly suicide rates, by gender

		Mean (±SD)	Maximum	Minimum
January				
	Men	1.768 (±0.107)	1.902	1.685
	Women	0.307 (±0.094)	0.454	0.209
February				
	Men	1.478 (±0.155)	1.649	1.268
	Women	0.391 (±0.062)	0.489	0.314
March				
	Men	1.442 (±0.260)	1.757	1.033
	Women	0.436 (±0.090)	0.506	0.314
April				
	Men	1.435 (±0.156)	1.594	1.214
	Women	0.342 (±0.068)	0.436	0.262
May				
	Men	1.576 (±0.304)	2.047	1.359
	Women	0.366 (±0.079)	0.454	0.279
June				
	Men	1.406 (±0.203)	1.631	1.141
	Women	0.356 (±0.083)	0.489	0.279
July				
	Men	1.424 (±0.159)	1.631	1.214
	Women	0.377 (±0.382)	0.436	0.332
August				
	Men	1.467 (±0.201)	1.757	1.214
	Women	0.387 (±0.100)	0.541	0.262
September				
	Men	1.474 (±0.146)	1.649	1.323
	Women	0.384 (±0.105)	0.524	0.279
October				
	Men	1.478 (±0.239)	1.757	1.178
	Women	0.304 (±0.065)	0.419	0.262
November				
	Men	1.529 (±0.127)	1.703	1.395
	Women	0.391 (±0.088)	0.471	0.279
December				
	Men	1.670 (±0.234)	1.975	1.468
	Women	0.419 (±0.083)	0.489	0.297

SD = standard deviation.

Odds ratios were calculated for two months of the year: January and December. Taking an a posteriori perspective and following our objectives and findings, we chose January and December because of the magnitudes of their differences between male and female suicide rates. In January, men had an odds ratio of 5.76 (95%CI = 4.59-7.22) in comparison to women. In December, men had an odds ratio of 3.99 (95%CI = 3.26-4.87) when compared to women.

**Figure 1:**
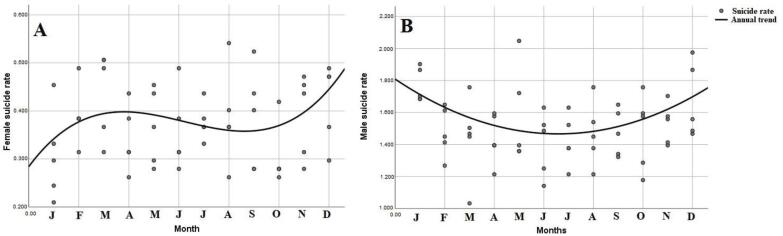
Monthly time trend analysis of male and female suicide rates: A) female; (B male. Months are shown by their initial letter, from January (left) to December (right).

We found that a quadratic regression model had the best fit to the male monthly suicide rate trend. Therefore, we showed that time, in months, had a statistically significant, nonlinear influence on male suicide rate ( Table 3 ). Suicide rates tended to be higher in the first and last months of the year, as shown by the concave shape of the curve. Throughout the year, male suicide rates tended to begin high, then decrease until mid-year (standardized β = -1.706), and finally increase until the end of the year (standardized β = 1.731). The monthly trend of male suicide can be represented by the curve shown in Figure 1 (B) .

**Table 3:** Time trend analysis of monthly suicide rates by gender

Variable/gender	Model (p-value * )	R^2^	B	Standardized β	Coefficient p-value
Men	Quadratic (< 0.001)	0.157	- 0.104	-1.706	**< 0.001**
			0.008	1.731	**< 0.001**
Women	Cubic (0.001)	0.095	0,071	2.957	**0.001**
			-0.013	-7.495	**< 0.001**
			0.001	4.818	**< 0.001**

Independent variable: months; dependent variable: suicide rates.

R^2^ = analysis of variance (ANOVA) R-squared.

* ANOVA p-value.

We found that cubic regression was the most suitable model for the women’s rate. Hence, monthly trend progression of female suicide rates can be divided into three parts: increasing rates in the first three months (standardized β = 2.957), then decreasing rates in the next five months (standardized β = - 7.495), and finally increasing rates in the last four months of the year (standardized β = 4.818), as shown in Table 3 and Figure 1 (A) .

**Figure 2:**
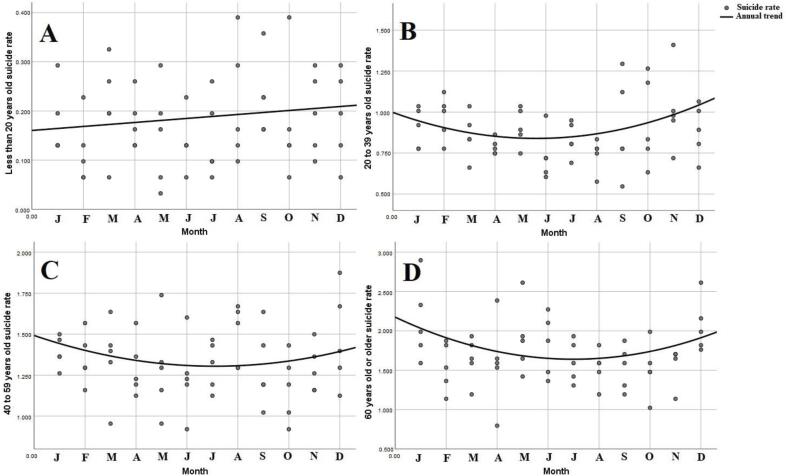
Monthly time series analysis of suicide by age range: A) less than 20 years old; B) 20 to 39 years old; C) 40 to 59 years old; D) 60 years or older. Months are shown by their initial letter, from January (left) to December (right).

Analyzing age range differences in suicide mortality, we found significant time trends for each range. For people less than 20 years old, we found that linear regression had the best fit. Therefore, suicide rates for people less than 20 years old tend to increase through the year (standardized β = 0.162), as shown in Table 4 and Figure 2 (A) , with a peak in December. Considering the other age ranges (20 years or older), we found that quadratic regression had the best fit for each of them. Thus, peaks tends to be in the first and last months of the year, as shown in Figure 2 (B), (C), and (D) . The most evident time trend was seen in people 60 years old or older, with both equation constants highly significant ( Table 4 ).

**Table 4:** Time trend analysis of monthly suicide rates by age ranges

Variable/age range	Model (p-value * )	R^2^	B	Standardized β	Coefficient p-value
Less than 20 yo	Linear (0.030)	0.026	0.004	0.162	**0.030**
From 20 to 39 yo	Quadratic (< 0.001)	0.107	-0.056	-1.045	**0.001**
			0.005	1.241	**< 0.001**
From 40 to 59 yo	Quadratic (0.045)	0.034	-0.053	-0.803	**0.014**
			0.004	0.756	**0.020**
60 yo or older	Quadratic (< 0.001)	0.097	-0.153	-1.356	**< 0.001**
			0.011	1.294	**< 0.001**

Independent variable: months; dependent variable: suicide rate.

R^2^ = ANOVA squared R; yo = years old.

* ANOVA p-value.

## Discussion

We evaluated seasonality, from a monthly perspective, and population features related to suicide mortality in RS, since studies have shown that this state appears to have the highest suicide rates in Brazil.^8 , 9^ Our study has three main findings. First, when compared to women, men had higher rates of suicide mortality in all age ranges and in all months of the year, and a higher risk of committing suicide. Second, older age ranges were associated with higher suicide rates, and people 60 years old or older had a higher risk of suicide when compared to those less than 20 years old. Third, men and women had different seasonal suicide rate patterns, mainly diverging in the first three months of the year. People in different age ranges also had different seasonal patterns, and those 60 years old or older showed the most evident trend. These findings are consistent with and complement existing literature nationally^9 , 14^ and globally.^15 – 17^

There are some possible explanations for the higher rates of completed suicide in men. First, the choice of method. It has been found that men are more lethal when they attempt suicide in comparison to women because they tend to use more violent methods, such as hanging and firearms.^18^ This is called the lethality explanation.^6^ Second, men appear to have more intent to die than women, and one possible explanation is that women tend to attempt suicide earlier on in the course of psychiatric morbidity.^19^

We found significant differences between genders and age ranges in monthly trends of suicide deaths. Men had a concave trend, in which January and December were the trending peaks of suicide rates. Conversely, women had trending peaks of suicide rates in March and December, and a trend to lower rates than men in January. Thus, considering risk, we found that men had a 5.76-fold higher risk of suicide than women in January and a 3.99-fold higher risk in December. Regarding age ranges, people less than 20 years old appeared to have a linear trend over the year, with the highest suicide rates occurring in December. However, age ranges over 20 years had a concave trend, with a monthly distribution similar to that for men. Therefore, their trending peak was in January and December, in late spring/early summer.

Over the years, researchers have found a seasonal association between suicide and time, mainly in temperate zone areas, where there are greater differences in climatic features throughout the year.^10 , 20 – 22^ In Italy, Preti^23^ reported differences in the seasonal distribution of suicide according to age. He found that young people showed a less evident asymmetrical distribution by season. In contrast, for people over 65 years old, clear asymmetry was observed in both genders, with a higher number of deaths in spring. In Iran, Veisani et al.^21^ described urban suicide rates with significant seasonal variations, with a peak in the spring and a trough in the winter. Seasonal differences were observed in all ages and both genders. In Brazil, Benedito-Silva et al.^10^ evaluated differences in suicide using data from eight different states. They found that annual suicide seasonality was only significant in the south of Brazil, with a peak in late spring and early summer, and found that the amplitude of the annual suicide pattern was associated with latitude. Also, Bando et al.^24^ described significant differences in seasons considering suicide rates, with differences present in spring, with higher rates, when compared to autumn, with lower rates. In Singapore, however, Parker et al.^25^ reported a weak seasonal distribution of suicide rates.

We analyzed a sample from the south of Brazil, where the climate is considered temperate subtropical and the four different seasons (summer, autumn, winter, and spring) are well defined, with warm summers and cold winters, differing from Brazil’s northern states.^26^ Therefore, our study complements the literature, since we found a significant time pattern in suicide rates and were able to specify these rates by gender and age ranges. Regarding gender, women had a mainly late spring/early summer, late summer/early autumn pattern, since there was an important decrease in suicide rates in January (mid-summer), with peaks in March and December. In contrast, Men had a late spring/early-to-mid-summer pattern (December and January). Considering age range, people over the age of 20 years had a pattern similar to that observed for men, with a trending peak in late spring and early summer. The trend was most evident for people aged 60 years or older. Individuals less than 20 years old, however, had one apparent peak, in late spring/early summer, with a less asymmetrical distribution. Except for the trend for women to have a low rate in January, these findings are similar to and coherent with existing literature.^10 , 21 , 23^

The effects of seasonality have not yet been well understood. In a systematic review, Turecki & Brent^27^ discussed the variability of associations of causes and risk factors according to gender, age, culture, etc. Among population-level risk factors, they described differences in suicide levels between societies that have been through social changes, such as disruption of traditions, and societies with high cohesion and common values. Economic crises that cause income reduction and unemployment have also been associated with higher rates of suicide, mainly in men. From this perspective, a hypothesis can be proposed regarding our findings and the correlation between annual distribution of suicide rates. As RS contributes almost 12% to Brazilian agriculture gross value added^28^ and agriculture contributes approximately 10% to RS’s GDP,^29^ climatic changes throughout the year can have an important impact on economic income for part of the population.

From a different perspective, the monthly trend in suicide rates has been associated with holidays in many countries. Traditional holidays, such as Christmas and Easter, were described as “death dips” for suicide rates, with lower mortality in the holiday period followed by increased deaths.^30^ Other studies in Europe did not find any decrease in suicide rate at Easter but reported an increase in the following week.^31 , 32^

Possible explanations were the desire to participate in special occasions or the transfer of suicidal feelings onto cultural events.^30^ In the Netherlands, Hofstra et al.^11^ described an interesting trend near Christmas. They showed a substantive decrease in mean daily suicide on December 25, followed by a substantive increase in mean daily suicide on December 27 and on January 1. This decrease in daily suicide near Christmas has also been described in Austria.^31^ On New Year’s Day, in contrast, suicide appeared to increase in different countries.^31 – 34^ Moreover, Carnival is an important holiday in Brazil that occurs in late February/early March. As far as we know, it has not been described as a factor associated with suicide in Brazil, but since our data showed a higher suicide rate in March for women, the effects of this holiday must be considered. Regarding our study, since we obtained monthly rather than daily data, we could not specify the influence of holidays on our dataset. However, the effects of holidays on suicide rates are one possible explanation for our monthly trends.

### Limitations

Our study has some limitations to be discussed. Our data were collected in an aggregate form, which restricts our capability of making correlations between variables. Moreover, much has been discussed about death underreporting in Brazil. There is a possibility of underreported data regarding deaths in the Brazilian Health Ministry databases because of the mortality notification system,^35 , 36^ but since this systemic error tends to be random, frequencies and proportions tend to be maintained.^14^ Suicide is a sensitive issue and registration is therefore complicated since it involves multiple medical and legal steps, depending on the country.^5^ In Brazil, the possibility of losing insurance and rights might have an important influence on notifications.^36 , 37^ According to the WHO, Brazil is on the list of countries with the best quality suicide data, with comprehensive vital registration and at least five years of data.^5^ Considering our limitations, further research is needed to better understand suicide rates, patterns, and correlations.

## Conclusion

This study has an important role in epidemiology and public policies regarding suicide. We showed that suicide in RS, Brazil, has visible monthly time trends and that trends vary between genders and age ranges. Also, we have brought knowledge about suicide numbers in the south of Brazil up to date, as we found that men and older people had higher rates of suicide than women and younger people, respectively. Using ratios, we found that men were at greater risk of committing suicide when compared to women, and that people aged 60 years or older were at higher risk of suicide in comparison to people less than 20 years old. We discussed the possible reasons for the monthly distribution of suicide along the year and possible reasons for higher rates of suicide among men, raising more questions and hypotheses about suicide features.
